# The Potential Economic Value of a *Trypanosoma cruzi* (Chagas Disease) Vaccine in Latin America

**DOI:** 10.1371/journal.pntd.0000916

**Published:** 2010-12-14

**Authors:** Bruce Y. Lee, Kristina M. Bacon, Diana L. Connor, Alyssa M. Willig, Rachel R. Bailey

**Affiliations:** 1 Public Health Computational and Operations Research (PHICOR), School of Medicine, University of Pittsburgh, Pittsburgh, Pennsylvania, United States of America; 2 Department of Biomedical Informatics, School of Medicine, University of Pittsburgh, Pittsburgh, Pennsylvania, United States of America; 3 Department of Epidemiology, Graduate School of Public Health, University of Pittsburgh, Pittsburgh, Pennsylvania, United States of America; Yale University, United States of America

## Abstract

**Background:**

Chagas disease, caused by the parasite *Trypanosoma cruzi (T. cruzi)*, is the leading etiology of non-ischemic heart disease worldwide, with Latin America bearing the majority of the burden. This substantial burden and the limitations of current interventions have motivated efforts to develop a vaccine against *T. cruzi*.

**Methodology/Principal Findings:**

We constructed a decision analytic Markov computer simulation model to assess the potential economic value of a *T. cruzi* vaccine in Latin America from the societal perspective. Each simulation run calculated the incremental cost-effectiveness ratio (ICER), or the cost per disability-adjusted life year (DALY) avoided, of vaccination. Sensitivity analyses evaluated the impact of varying key model parameters such as vaccine cost (range: $0.50–$200), vaccine efficacy (range: 25%–75%), the cost of acute-phase drug treatment (range: $10–$150 to account for variations in acute-phase treatment regimens), and risk of infection (range: 1%–20%). Additional analyses determined the incremental cost of vaccinating an individual and the cost per averted congestive heart failure case. Vaccination was considered highly cost-effective when the ICER was ≤1 times the GDP/capita, still cost-effective when the ICER was between 1 and 3 times the GDP/capita, and not cost-effective when the ICER was >3 times the GDP/capita. Our results showed vaccination to be very cost-effective and often economically dominant (i.e., saving costs as well providing health benefits) for a wide range of scenarios, e.g., even when risk of infection was as low as 1% and vaccine efficacy was as low as 25%. Vaccinating an individual could likely provide net cost savings that rise substantially as risk of infection or vaccine efficacy increase.

**Conclusions/Significance:**

Results indicate that a *T. cruzi* vaccine could provide substantial economic benefit, depending on the cost of the vaccine, and support continued efforts to develop a human vaccine.

## Author Summary

The substantial burden of Chagas disease, especially in Latin America, and the limitations of currently available treatment and control strategies have motivated the development of a *Trypanosoma cruzi* (*T. cruzi*) vaccine. Evaluating a vaccine's potential economic value early in its development can answer important questions while the vaccine's key characteristics (e.g., vaccine efficacy targets, price points, and target population) can still be altered. This can assist vaccine scientists, manufacturers, policy makers, and other decision makers in the development and implementation of the vaccine. We developed a computational economic model to determine the cost-effectiveness of introducing a *T. cruzi* vaccine in Latin America. Our results showed vaccination to be very cost-effective, in many cases providing both cost savings and health benefits, even at low infection risk and vaccine efficacy. Moreover, our study suggests that a vaccine may actually “pay for itself”, as even a relatively higher priced vaccine will generate net cost savings for a purchaser (e.g., a country's ministry of health). These findings support continued investments in and efforts toward the development of a human *T. cruzi* vaccine.

## Introduction

Chagas disease (American trypanosomiasis), caused by the parasite *Trypanosoma cruzi (T. cruzi)*, is a leading etiology of non-ischemic heart disease worldwide [1] and has a substantial impact on Latin America, resulting in an estimated 750,000 productive life years lost and 1.2 billion dollars lost annually [2], [3], [4]. Chagas disease has three established phases: acute, indeterminate, and chronic. While acute disease is primarily asymptomatic, cases often transition to the chronic phase and clinically manifest as cardiomyopathy and subsequent congestive heart failure (CHF) decades after infection [1], [5], [6]. Furthermore, those who develop Chagas-related CHF have poorer prognoses and higher mortality rates than those with other CHF etiologies [2].

The substantial burden of Chagas disease and the limitations of current interventions have motivated efforts to develop a vaccine against *T. cruzi*. Although currently available drugs (benznidazole and nifurtimox) are moderately efficacious when administered during the acute phase, they have been minimally successful in treating chronic infection [7], [8], [9]. Low rates of symptomatic acute illness limit the utilization (and thus, the benefit) of these drugs [5], [8]. The lack of an available vaccine has left insecticide spraying for T. *cruzi* vectors (reduvidae insects) as the primary control strategy. However, implementing successful mass spraying can be challenging [10], [11]. Mass spraying requires both repeated and consistent reapplication, which in turn necessitates funding, personnel, and equipment. Due to a lack of national-level funding, local communities may not have the resources to maintain spraying. Furthermore, increased use of insecticides has elicited resistance among vectors in Argentina and Bolivia and may lead to untoward health effects for humans [11].

Several *T. cruzi* vaccine candidates have demonstrated protective effects against challenge in mouse models and offer promise for the future development of a human vaccine [12], . Much attention has focused on DNA vaccines, consisting of one or more antigen-coding plasmids, which may provide sufficient protection without the possibility of reverting back to the infectious form [12].

Understanding the potential economic and health benefits of a *T. cruzi* vaccine could help guide vaccine investment, development, targeting, and implementation, thereby assisting vaccine scientists, manufacturers, policy makers, and other decision makers. It can be helpful to construct economic models early in a vaccine's development, before key decisions about the vaccine are made and while important aspects of the vaccine can still be altered [14]. We developed a computer model to evaluate the economic value of a *T. cruzi* vaccine for the control of Chagas disease in Latin America. Different scenarios helped determine the effects of varying various key vaccine characteristics such as vaccine efficacy (to guide development), vaccine cost (to help set future price points), and risk of infection (to identify appropriate target populations).

## Methods

### Model Structure

**Figure 1:**
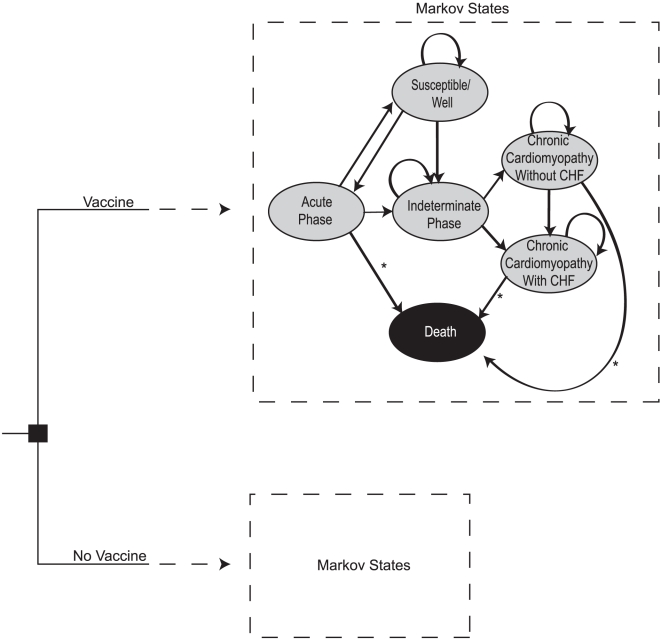
Model Markov states. * Arrows represent states where Chagas-related death could occur. Non-Chagas-related death could occur during any state.

We constructed a Markov decision analytic computer simulation model to assess the potential cost-effectiveness of a *T. cruzi* vaccine in Latin America using TreeAge Pro 2009 (TreeAge Software, Williamstown, Massachusetts). The model assumed the societal perspective and evaluated the economic value of vaccination versus no vaccination of a cohort of children <1 year of age to prevent *T. cruzi* infection and Chagas disease. Each cycle length was one year. Figure 1 illustrates the general model structure, including the following six Markov states of the disease model and an individual's possible transitions between states:

- Susceptible/Well: The individual was healthy and uninfected during this year.
- Acute Phase: The individual was actually infected with *T. cruzi*. Infected individuals only remain in this state for 1 year.
- Indeterminate Phase: The latent phase of disease subsequent to the acute phase. Those who became infected could remain here for 10 years or more before transitioning to a chronic form of Chagas disease, or could have remained in this phase for life.
- Chronic Cardiomyopathy without CHF: Cardiac abnormalities existed, but CHF had not developed. Cases that entered into this state then had an annual probability of progressing to CHF.
- Chronic Cardiomyopathy with CHF: Individual had CHF.
- Death: Death occurred as a result of either Chagas disease (acute or chronic) or other causes unrelated to *T. cruzi* infection.

All Markov states were mutually exclusive. Individuals entered the model at age 0 and began in the ‘Susceptible/Well’ state (i.e., well with no prior history of infection). Each passing year, the individual either remained in the same state or transitioned to another state. The individual continued in the model until he or she entered the Death state from (1) acute/symptomatic infection, (2) cardiomyopathy with or without CHF, or (3) mortality unrelated to *T. cruzi* infection, where they remained for the remainder of the trial [5], [6].

**Figure 2:**
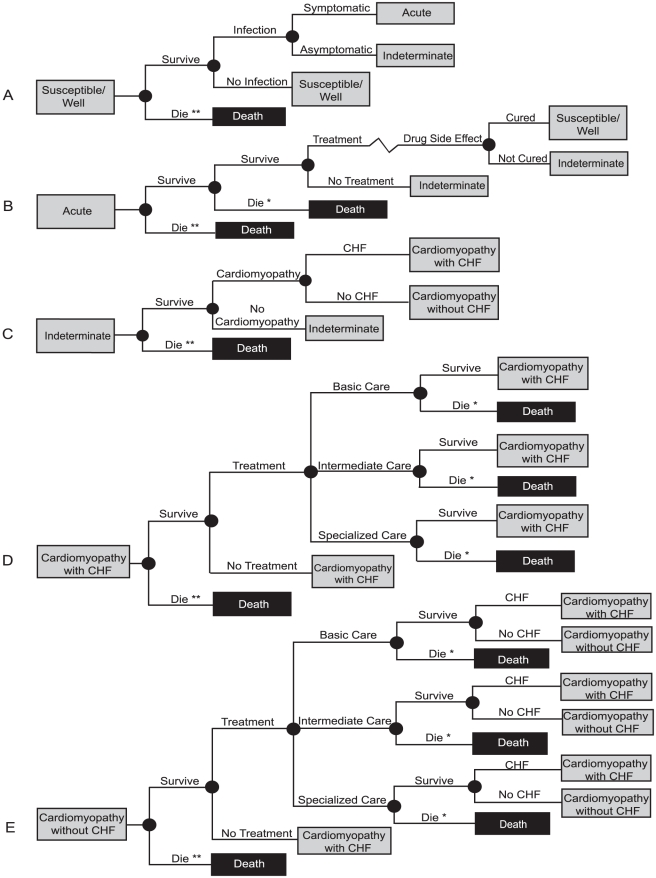
Markov state subtrees. A) Susceptible/Well B) Acute Phase C) Indeterminate Phase D) Cardiomyopathy with CHF E) Cardiomyopathy without CHF. * Death resulting from Chagas infection ** Death from non-Chagas related causes.

Figure 2a–e shows the possible paths an individual could have traveled through after entering each Markov state. After entering the ‘Susceptible/Well’ state, an individual had probabilities of becoming infected (symptomatic or asymptomatic infection), dying of unrelated causes, or remaining uninfected. Only symptomatic cases had a probability of seeking *T. cruzi* treatment during the acute phase. Consistent with standard medical operating procedure, developing severe side effects resulted in discontinuation of drug treatment and therefore eliminating any chance the treatment could be successful. Asymptomatic cases did not receive treatment and proceeded directly to the indeterminate (latent) disease phase. The reported time from acute illness to development of chronic cardiac manifestations in the literature ranged from 10 to 30 years; infected individuals in the model therefore had to stay in the indeterminate phase for at least 10 cycles (years) before having an annual probability of developing cardiomyopathy (with or without CHF) [5], [6], [15], [16], [17]. Those who sought a form of treatment for chronic infection once continued to do so throughout the remainder of their life span.

Each simulation run sent 1,000 individuals through the model 1,000 times each for a total of 1,000,000 individual realizations. For each simulation run, the following equation computed the incremental cost-effectiveness ratio (ICER), or cost per disability-adjusted life year (DALY) avoided, through vaccination:
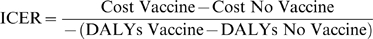
Quantification of DALY decrements for vaccinating and not vaccinating included the years lost due to disability from Chagas (YLD) as well as the years of life lost as a result of Chagas-related mortality (YLL). Costs included both direct and indirect costs (such as productivity loss) that resulted from becoming a Chagas case. The cost-effectiveness of vaccination for each scenario was evaluated using the GDP per capita of Colombia ($5,048.41), as it represented the approximate average GDP per capita for all of Latin America [18], [19]. Vaccination was considered highly cost-effective if the ICER value was $5,048.41 per DALY avoided or less. Scenarios that yielded an ICER of $15,145.23 per DALY (3 times the GDP per capita) avoided or less still indicated that vaccination was cost-effective, while ICERs greater than 3 times the GDP per capita indicated that vaccination was not a cost-effective strategy. The cost of vaccinating an individual was calculated by comparing the average cost accrued on the vaccine arm of the model to the average cost accrued on the no vaccine arm over the 1 million trials for each simulation. The cost per avoided congestive heart failure (CHF) case was calculated by dividing the cost difference between the vaccine and no vaccine arm for the entire cohort by the total number of CHF cases avoided on the vaccine arm/branch (compared to the no vaccine branch) for the simulation.

### Data Inputs

Table 1 shows the cost, probability, and DALY model input values and their corresponding sources. The probability of an acute case being symptomatic in the model was 1%. Based on a report of treatment-seeking behavior for febrile illness, the probability that a symptomatic individual sought treatment was 34% [5], [8], [20]. For treatment costs, life expectancy, and crude mortality rates, data from Colombia, where Chagas prevalence is high, served as a proxy for Latin America [19]. World Health Organization (WHO) sources provided disability weights for cardiomyopathy with and without CHF as well as crude mortality rates (0–11 mos, 1.65%; 1–4 yrs, 0.32%; 5–9 yrs, 0.15%; 10–14 yrs, 0.18%; 15–19 yrs, 0.55%; 20–24 yrs, 0.97%; 25–29 yrs, 1.08%; 30–34 yrs, 1.02%; 35–39 yrs, 1.02%; 40–44 yrs, 1.12%; 45–49 yrs, 1.58%; 50–54 yrs, 2.11%; 55–59 yrs, 3.12%; 60–64 yrs, 4.99%; 65–69 yrs, 9.07%; 70–74 yrs, 14.66%; 75–79 yrs, 23.85%; 80–84 yrs, 30.62%; 85–89 yrs, 40.46%; 90–94 yrs, 51.70%; 95–99 yrs, 64.50%; 100 yrs and older, 100%) and life expectancies (0–11 mos, 75.5 yrs; 1–4 yrs, 75.8 yrs; 5–9 yrs, 72 yrs; 10–14 yrs, 67.1 yrs; 15–19 yrs, 62.2 yrs; 20–24 yrs, 57.5 yrs; 25–29 yrs, 53.1 yrs; 30–34 yrs, 48.6 yrs; 35–39 yrs, 44.1 yrs; 40–44 yrs, 39.5 yrs; 45–49 yrs, 35 yrs; 50–54 yrs, 30.4 yrs; 55–59 yrs, 26 yrs; 60–64 yrs, 21.8 yrs; 65–69 yrs, 17.8 yrs; 70–74 yrs, 14.3 yrs; 75–79 yrs, 11.4 yrs; 80–84 yrs, 9.2 yrs; 85–89 yrs, 7.1 yrs; 90–94 yrs, 5.2 yrs; 95–99 yrs, 3.6 yrs; 100 yrs and older, 2.5yrs) [21], [22].

**Table 1:** Model inputs.

Variable	Baseline Value	Source
**PROBABILITIES**		
Benznidazole Cure Rate Acute Phase	0.65^a^	[5], [26], [28]
Drug Side Effects	0.05	[28]
Mortality Acute Phase	0.05^b^	[5], [6]
Annual Rate of Progression to Chronic Phase	0.02	[15]
Cardiomyopathy	0.25^c^	[5]
Seek Treatment if Cardiomyopathy	0.35	[6]
CHF if Cardiomyopathy	0.04	[6]
Mortality CHF	0.30	[6]
Mortality No CHF	0.04	[6]
**DISABILITY WEIGHTS**		
Cardiomyopathy CHF	0.27	[22]
Cardiomyopathy No CHF	0.06	[22]
**COSTS (US$)**		
Palliative Care Only CHF^d^	42	[16]
Palliative Care Only No CHF	23	[16]
Basic Care CHF^e^	61	[16]
Basic Care No CHF^f^	55	[16]
Intermediate Care CHF^g^	310	[16]
Intermediate Care No CHF	224	[16]
Specialized Care CHF^h^	9,530	[16]
Specialized Care No CHF	4,360	[16]
Indeterminate Phase	105	[6]

a Uniform distribution with a lower limit of 0.5 and an upper limit of 0.8.

b Triangular distribution with a lower limit of 0.01 and an upper limit of 0.1.

c Uniform distribution with a lower limit of 0.2 and an upper limit of 0.3.

d Refers to cases only seeking care to minimize their symptoms, not to prevent or delay progression of disease.

e Consisted of 3 general practitioner visits and 0.58 days in the hospital.

f Consisted of 5.53 general practitioner visits and 0.25 days in the hospital.

g Included outpatient visits, diagnostics, medicine, and hospitalization costs.

h Included outpatient care, hospitalization, and hospital procedures such as electrocardiography, x-rays, and surgery.

### Sensitivity Analyses

Sensitivity analyses evaluated the impact of varying key model parameters such as vaccine cost (ranging from a low of $0.50 up to the cost at which the vaccine was no longer cost-effective), vaccine efficacy in preventing infection (range: 25% to 75%), the cost of acute-phase drug treatment (range: $10–$150 to account for variations in acute-phase treatment regimens), and risk of infection (range: 1%–20%) [23]. As many studies report a cost associated with routine surveillance (i.e., clinic visits, radiographs, electrocardiograms, and laboratory tests) during the indeterminate phase, additional sensitivity analyses varied the probability that an individual accrued a cost while in this phase from 25%–75% [6], [24]. Probabilistic sensitivity analyses determined the effects of simultaneously varying parameter values across their respective ranges.

## Results

### Overall Impact

Results suggest that a *T. cruzi* vaccine would be cost-effective across a wide range of vaccine prices and efficacies and *T. cruzi* infection risks. In fact, in many cases, a *T. cruzi* vaccine could actually save costs (i.e., that would be associated with treating the disease) in addition to providing health benefits. Vaccination remained cost-effective even up to a vaccine price of $75 when infection risk was 5% and vaccine efficacy was greater than 50% and up to a vaccine price of $200 when the vaccine efficacy was 75% or greater.

### Cost-Effectiveness

The majority of our modeled scenarios demonstrated vaccination to be very cost-effective, and in many cases highly cost-effective, especially with lower vaccine price points, compared to no vaccination. Table 2 indicates the incremental cost-effectiveness ratio (ICER) of Chagas vaccination at various vaccine costs, infection risks and vaccine efficacy rates. When the total vaccine cost was less than $5, vaccination was highly cost-effective across all scenarios of infection risk (1%, 5%, 10% and 20%) and vaccine efficacy (25%, 50%, and 75%). Increasing the vaccine price to $10 alters the vaccine strategy to cost-effective across all scenarios, and finally when the vaccine cost is $20 and vaccine efficacy is 25%, some scenarios become cost-ineffective. However, when the vaccine efficacy is greater than 50%, vaccination remains cost-effective until a $50 vaccine price point at 1% infection rate, a $75 vaccine price point at 5% infection rate, and a $100 vaccine price point at 10% infection rate. Vaccination remains cost-effective at a $200 price point when the efficacy is greater than 50% and infection risk is 20%. The model was fairly robust and displayed minimal sensitivity to variation in treatment costs for acute infection and the probability of an indeterminate phase-associated cost.

**Table 2:** The incremental cost-effectiveness ratio (ICER) of Chagas vaccination in US dollars.

		Risk of *T. cruzi* Infection			
	*Vaccine Efficacy*	1%	5%	10%	20%
**Vaccine Cost = $0.50**					
	*25%*	**Vaccinate**	**Vaccinate**	**Vaccinate**	**Vaccinate**
	*50%*	**Vaccinate**	**Vaccinate**	**Vaccinate**	**Vaccinate**
	*75%*	**Vaccinate**	**Vaccinate**	**Vaccinate**	**Vaccinate**
**Vaccine Cost = $1**					
	*25%*	**Vaccinate**	**Vaccinate**	**Vaccinate**	**Vaccinate**
	*50%*	**Vaccinate**	**Vaccinate**	**Vaccinate**	**Vaccinate**
	*75%*	**Vaccinate**	**Vaccinate**	**Vaccinate**	**Vaccinate**
**Vaccine Cost = $5**					
	*25%*	**1,335**	**Vaccinate**	**Vaccinate**	**Vaccinate**
	*50%*	**Vaccinate**	**Vaccinate**	**Vaccinate**	**Vaccinate**
	*75%*	**Vaccinate**	**Vaccinate**	**Vaccinate**	**Vaccinate**
**Vaccine Cost = $10**					
	*25%*	**5,485**	**2,526**	**Vaccinate**	**Vaccinate**
	*50%*	**1,331**	**Vaccinate**	**Vaccinate**	**7,178**
	*75%*	**800**	**Vaccinate**	**Vaccinate**	**Vaccinate**
**Vaccine Cost = $20**					
	*25%*	**10,111**	**1,208**	Don't Vaccinate	**1,143**
	*50%*	**7,541**	**205**	**Vaccinate**	**Vaccinate**
	*75%*	**5,629**	**Vaccinate**	**Vaccinate**	**Vaccinate**
**Vaccine Cost = $30**					
	*25%*	Don't Vaccinate	**5,358**	**1,520**	**6,967**
	*50%*	**10,849**	**746**	**Vaccinate**	**Vaccinate**
	*75%*	**8,987**	**Vaccinate**	**Vaccinate**	**Vaccinate**
**Vaccine Cost = $50**					
	*25%*	Don't Vaccinate	21,787	30,651	**5,031**
	*50%*	23,012	**3,201**	**1,007**	**898**
	*75%*	21,234	**1,319**	**Vaccinate**	**Vaccinate**
**Vaccine Cost = $75**					
	*25%*	221,657	35,065	**14,051**	17,806
	*50%*	26,492	**14,614**	**4,097**	**5,316**
	*75%*	55,203	**6,872**	**713**	**176**
**Vaccine Cost = $100**					
	*25%*	Don't Vaccinate	45,307	46,693	34,017
	*50%*	450,040	36,600	**8,757**	**5,263**
	*75%*	20,639	**3,091**	**4,452**	**3,875**
**Vaccine Cost = $200**					
	*25%*	122,109	71,062	26,972	36,640
	*50%*	Don't Vaccinate	58,412	50,385	**8,743**
	*75%*	46,471	**12,536**	**8,247**	**5,485**

Bold numbers indicate a cost-effective strategy.

### Budget Impact Analysis

Table 3 shows that vaccinating an individual can actually be cost savings under many explored circumstances. Negative values in the table indicate that vaccinating an individual resulted in cost savings (i.e., there was actually net monetary gain from vaccinating an individual). For example, when a $1 vaccine was only 25% efficacious and the risk of infection was 1%, vaccinating an individual would on average save $1.52. At infection rates as low as 1%, vaccination was cost-saving up to a $5 vaccine price point as long as the vaccine remained at least 50% efficacious, with savings overall ranging from −$0.07 to −$81.26 per vaccinated individual. Vaccination remained cost-saving for all scenarios under a $10 vaccine price point, when the risk of infection was 5% or greater, averting the most cost (−$81) per vaccinee when vaccine price was $1, efficacy was 75%, and infection risk was 20%. Administering vaccine only resulted in net cost ($2.44–$7.26) at low infection risk (1%) and vaccine price point of $10 for the entire range of evaluated vaccine efficacies. The cost benefit persisted when the vaccine efficacy was 50% at vaccine costs of $20–$30 when infection risk was greater of equal to 10%. When the vaccine was 75% efficacious, a cost savings exists for vaccine prices of ≤$50. For a vaccine cost of $200, the incremental cost of vaccinating an individual ranged from $111.03 (20% infection rate, 75% vaccine efficacy) to $197.36 (1% infection rate, 25% vaccine efficacy).

**Table 3:** The incremental cost of vaccinating an individual ($US).

		Risk of *T. cruzi* Infection			
	*Vaccine Efficacy*	1%	5%	10%	20%
**Vaccine Cost = $1**					
	*25%*	**−1.52**	**−9.26**	**−15.61**	**−18.49**
	*50%*	**−4.29**	**−21.92**	**−31.24**	**−50.02**
	*75%*	**−7.10**	**−38.07**	**−55.04**	**−81.26**
**Vaccine Cost = $5**					
	*25%*	2.21	**−5.99**	**−10.78**	**−15.65**
	*50%*	**−0.07**	**−16.47**	**−33.73**	**−40.88**
	*75%*	**−4.08**	**−31.31**	**−54.09**	**−78.27**
**Vaccine Cost = $10**					
	*25%*	7.26	**−0.94**	**−7.21**	**−10.45**
	*50%*	4.54	**−12.97**	**−28.66**	**−36.90**
	*75%*	2.44	**−26.71**	**−51.38**	**−76.66**
**Vaccine Cost = $20**					
	*25%*	17.53	10.28	3.73	1.98
	*50%*	15.47	1.18	**−13.28**	**−22.20**
	*75%*	13.02	**−11.52**	**−33.08**	**−54.50**
**Vaccine Cost = $30**					
	*25%*	27.57	21.37	13.99	11.96
	*50%*	25.80	10.54	**−3.23**	**−10.45**
	*75%*	23.35	**−2.58**	**−20.68**	**−48.44**
**Vaccine Cost = $50**					
	*25%*	47.52	40.78	36.18	7.96
	*50%*	45.10	28.77	17.91	33.27
	*75%*	42.54	17.79	**−1.28**	**−24.04**
**Vaccine Cost = $75**					
	*25%*	72.91	64.70	60.51	55.47
	*50%*	70.64	54.00	43.79	31.81
	*75%*	68.30	43.62	20.78	3.45
**Vaccine Cost = $100**					
	*25%*	97.50	90.26	84.13	81.40
	*50%*	95.58	80.67	68.17	60.08
	*75%*	93.37	67.85	45.09	28.40
**Vaccine Cost = $200**					
	*25%*	197.36	190.82	184.64	181.15
	*50%*	194.71	180.03	165.09	154.43
	*75%*	193.28	166.69	148.32	111.03

Incremental costs refer to the difference between the cost of vaccinating and the cost of not vaccinating. Negative values indicate net cost savings with vaccination; positive values indicate net costs with vaccination.

### Cost per CHF Case Averted

Table 4 displays the cost per CHF case averted by vaccination. As can be seen, since the vaccine in most cases is cost savings, there is actually net savings per CHF case averted. The greatest cost savings per CHF case avoided were seen at the lowest vaccine cost ($1) and the lowest infection rates (1%). Under every scenario with a vaccine cost of ≤$10 (regardless of indeterminate phase-cost association or vaccine efficacy) and infection rates of 5% or above, the cost per CHF case averted was negative, indicating a cost savings per case avoided through vaccination. When infection rates were 1% and vaccine cost increased to $10, the cost per avoided CHF case was $50, 883 at 75% vaccine efficacy. At lower efficacies (50% and 25%), cost per CHF case averted increased to $126,009 and $660,350, respectively. At a vaccine price point of $5, vaccinating to prevent a case of CHF ranged from costing over $100,000 to saving over $100,000, depending on vaccine efficacy.

**Table 4:** The cost per avoided case of congestive heart failure (CHF) ($US).

		Risk of *T. cruzi* Infection			
	*Vaccine Efficacy*	1%	5%	10%	20%
**Vaccine Cost = $1**					
	*25%*	**−108,779**	**−132,304**	**−130,069**	**−81,435**
	*50%*	**−195,117**	**−171,231**	**−135,221**	**−100,237**
	*75%*	**−154,287**	**−186,595**	**−152,040**	**−115,263**
**Vaccine Cost = $5**					
	*25%*	234,280	**−117,542**	**−91,333**	**−66,319**
	*50%*	**−2,743**	**−145,784**	**−156,898**	**−96,642**
	*75%*	**−110,259**	**−166,543**	**−146,197**	**−110,397**
**Vaccine Cost = $10**					
	*25%*	660,350	**−19,480**	**−63,273**	**−41,302**
	*50%*	126,009	**−102,932**	**−118,409**	**−78,352**
	*75%*	50,883	**−157,108**	**−138,875**	**−108,278**
**Vaccine Cost = $20**					
	*25%*	1,252,352	151,155	31,876	8,486
	*50%*	967,156	10,071	**−57,757**	**−44,047**
	*75%*	419,868	**−61,587**	**−93,459**	**−72,470**
**Vaccine Cost = $30**					
	*25%*	2,121,101	403,277	123,817	41,973
	*50%*	1,032,053	83,686	**−13,118**	**−20,900**
	*75%*	631,111	**−13,236**	**−57,275**	**−69,105**
**Vaccine Cost = $50**					
	*25%*	4,751,919	608,643	282,693	18,599
	*50%*	1,503,296	241,724	74,020	149,209
	*75%*	1,519,255	102,249	**−3,714**	**−33,620**
**Vaccine Cost = $75**					
	*25%*	4,860,652	1,043,606	521,635	224,576
	*50%*	3,360,131	409,098	207,532	58,802
	*75%*	1,751,278	241,000	55,279	4,612
**Vaccine Cost = $100**					
	*25%*	8,863,707	1,920,387	657,249	347,864
	*50%*	3,540,061	783,224	333,905	129,760
	*75%*	2,593,484	387,727	122,528	40,520
**Vaccine Cost = $200**					
	*25%*	14,097,245	2,981,599	1,538,697	846,508
	*50%*	8,465,638	1,636,654	657,726	307,633
	*75%*	5,368,789	963,519	429,911	170,284

Negative values indicate net cost savings with vaccination; positive values indicate net costs with vaccination.

## Discussion

Our results support further development and future implementation of a *T. cruzi* vaccine and have several implications for vaccine policy makers, developers, and manufacturers. A *T. cruzi* vaccine could not only decrease infection burden, but also severe cardiac disease in Latin America. Our results suggest that vaccination would be highly cost-effective (and in some cases, economically dominant) over a wide range of infection rates, treatment-seeking behaviors, and vaccine costs. Such findings imply that vaccination may be beneficial even in areas with low risk of infection or in endemic areas that are improving (i.e., decreasing infection risk) over time. Demonstrating that the vaccine could provide net cost savings even at relatively higher vaccine price points (such as $50) could encourage manufacturers to pursue development and eventual commercialization of the vaccine. Additionally, as our analysis suggests that the target efficacy window for a vaccine can be fairly wide; scientists do not necessarily have to design the near “perfect” vaccine that confers protection close to 100% in order to establish economic value. Even vaccines that offer lower levels of protection can be valuable, especially at lower vaccine prices. Moreover, our study suggests that a *T. cruzi* vaccine may actually “pay for itself”: even a relatively higher priced vaccine of $50 can generate net cost savings for a purchaser (e.g., a country's ministry of health).

As our results demonstrate, vaccine price helps drive the net costs and cost-effectiveness of a *T. cruzi* vaccine. A vaccine that costs $30 or less is cost-effective under most explored conditions. However, a vaccine that costs $100 or more is cost-effective under a much more limited set of circumstances. Our results may help decision-makers map out the appropriate prices for a given situation.

A *T. cruzi* vaccine could affect the epidemiology of cardiac disease in endemic populations and countries. As management of cardiomyopathy and CHF are associated with intensive and costly medical care, preventing such outcomes would likely alleviate a substantial burden on the healthcare system. A study conducted by Mendez *et al.* reported that 20% of all cardiovascular disease patients seen in a particular Brazilian health institution had Chagas-related cardiomyopathy, and suggests that unresolved Chagas infection accounts for nearly 30% of all CHF cases in Brazil [25]. Additional literature suggests that a similar proportion of CHF cases (20%) in Colombia result from Chagas disease [2]. Chagas disease may play an even larger role in causing cardiac disease in other parts of Latin America.

Limitations of current alternatives have spurred efforts to develop a *T. cruzi* vaccine. Although drugs exist for the treatment of Chagas disease, they have many complications and are associated with long treatment regimens and low cure rates when administered after the acute disease phase [26]. DNA vaccines have recently shown promise against several protozoan parasitic diseases, such as malaria, leishmaniasis, and Chagas disease, as they have proven to be safe and elicit a complete immune response. Furthermore, their thermo-stability and affordability may make them highly practical for use in resource poor settings where these diseases are endemic. While this technology has yet to yield an efficacious Chagas vaccine for the human population, the development and licensure of DNA vaccines against West Nile and infectious hematopoietic necrosis viruses in animals give promise for a future human vaccine [12]. Certainly bringing a *T. cruzi* vaccine to market would involve surmounting a variety of scientific hurdles, but our study suggests that surmounting such obstacles could be very worthwhile [12].

In developing our model, we attempted to remain conservative about the benefits of a vaccine. The actual costs of diagnosing and treating CHF may be much higher than the numbers used. In addition, CHF could have substantial repercussions on an individual's life which were not captured by the model. The model assumed that individuals were otherwise healthy, but the addition of co-morbidities or co-infection with other pathogens such as the human immunodeficiency virus (HIV) could worsen outcomes for individuals infected with *T. cruzi*. Moreover, our model did not consider how the vaccine may reduce the transmission of *T. cruzi* by reducing available human hosts. The risk of infection may be higher than reported, as many cases go undiagnosed. Our range of infection probabilities came from Global Health Statistics reports but may not include regions and populations with higher risk [2], [25], [27]. Some studies have reported the direct progression from acute to chronic disease as well as sudden death due to cardiac complications during the indeterminate phase; however, these possibilities were difficult to measure accurately and therefore not incorporated into the model parameters [5].

### Limitations

All computer models are simplifications of real world scenarios and therefore cannot capture all possible outcomes of Chagas disease or the efficacy of concurrent existing regional control methods. Our model focuses on the individual rather than a population and therefore, does not explicitly represent herd immunity. However, increasing herd immunity could decrease Chagas risk, and our study demonstrates how changes in Chagas disease risk would affect the vaccine's economic value. Additionally, this model cannot account for the variation in high risk exposure resulting from factors such as environmental conditions or individual behaviors across Latin America. Although model assumptions and data inputs were drawn from an extensive review of the literature, sources may vary in quality and input values may not hold under all conditions.

### Conclusion

Our model suggests that introducing a *T. cruzi* vaccine to Latin America would provide economic value. Such a vaccine could be highly cost-effective and many cases could be economically dominant, providing both cost savings and health benefits. Even a vaccine with fairly low efficacy (25%) can provide cost savings. Moreover, a vaccine could be cost-effective even at relatively low infection risks (1%). Such findings support continued efforts to develop a human vaccine against *T. cruzi* to help reduce the significant burden of Chagas disease.
